# Pancreatic neuroendocrine tumor with metastasis to the spleen: a case report

**DOI:** 10.1186/s12885-016-3020-8

**Published:** 2017-01-09

**Authors:** Yasunaru Sakuma, Yoshikazu Yasuda, Naohiro Sata, Yoshinori Hosoya, Atsushi Shimizu, Hirofumi Fujii, Daisuke Matsubara, Noriyuki Fukushima, Atsushi Miki, Misato Maeno, Alan Kawarai Lefor

**Affiliations:** 1 Department of Surgery, Jichi Medical University, Tochigi, Japan; 2 Department of Oncology, Jichi Medical University, Tochigi, Japan; 3 Department of Pathology, Jichi Medical University, Tochigi, Japan

**Keywords:** Pancreatic neuroendocrine tumor, Spleen metastasis, Liver metastasis, Somatostatin, Radiofrequency ablation, Case report

## Abstract

**Background:**

Long-term term survival in patients with pancreatic neuroendocrine tumors has been reported, even in patients with metastatic disease. Metastases to the spleen are extremely rare, but have been reported from a number of primary malignancies, such as breast cancer, lung cancer, melanoma and ovarian cancer. This is the first report of a splenic metastasis from a primary pancreatic neuroendocrine tumor.

**Case presentation:**

The patient presented as a 53 years old white male with anemia and fatigue. Physical examination revealed a left upper quadrant fullness and computed tomography showed a 24 cm left upper quadrant mass with multiple liver metastases, splenomegaly and a 1 cm mass in the spleen. Resection of the primary pancreatic tumor (T4N0M1) was accompanied by gastrectomy, splenectomy and resection of adherent bowel. The spleen contained a metastatic lesion 1.0 cm in diameter, consistent with a primary neuroendocrine tumor of the pancreas. This operation was followed 8 months later, by delayed resection of liver metastases. The patient receives monthly administration of somatostatin long-acting analogue and has undergone several ablations of liver lesions with percutaneous radiofrequency ablation as well as a second liver resection. The patient is alive seven years after initial presentation, with no evidence of disease on imaging studies.

**Conclusions:**

This is the first report of a splenic metastasis from a primary pancreatic neuroendocrine tumor. The patient initially presented with synchronous multiple liver metastases and a single splenic metastasis. After resection of the primary tumor and spleen, the patient has undergone aggressive cytoreductive surgery/ablation of liver lesions and somatostatin therapy with resulting long-term survival.

## Background

Neuroendocrine tumors occur throughout the body, and have a variety of presentations and clinical characteristics. Pancreatic neuroendocrine tumors (pNETs) are particularly rare, representing only 1-2% of all pancreatic neoplasms, but the treatment of these lesions continues to evolve with improving results. NETs are classified separately from carcinoid tumors, in part based on location [1–3]. The clinical presentation of NETs primarily depends on whether they are functional or non-functional, and if they are functional, depending on what hormone is being produced [2]. Compared with NETs from other origins, patients with NETs of gastrointestinal origin are more likely to have metastases at the time of presentation [4]. The prognosis of patients with these tumors depends on a number of factors, but liver metastases are very common, being present at the time of presentation in up to 65% of patients, and are often associated with a poorer prognosis [2]. Biologic factors related to the tumor (Ki-67 index and mitotic index) have also been shown to be important and are incorporated into the 2010 WHO grading scheme for these lesions [2, 3, 5]. Prolonged survival, even in the presence of liver metastases has been reported with aggressive treatment [1, 4].

**Table 1:** Timeline of care

Prior History
• -9 years: Left ulnar nerve decompression • -3 years: Roux-en-Y gastric bypass for obesity with resolution of previously untreated Type 2 Diabetes Mellitus
Diagnosis and Interventions
• Anemia, fatigue, left upper quadrant fullness on physical examination, serum hemoglobin 7 mg/dl • CT scan showed a 24 cm left upper quadrant mass with multiple liver metastases and a single 1 cm spleen metastasis • Laparoscopic liver biopsy: read as hepatocellular carcinoma vs. hepatoid carcinoma • +1 month: Serum hemoglobin 4.0 g/dl, surgery for completion gastrectomy, splenectomy, small bowel resection, colon resection, and distal pancreatectomy, complicated by intra-abdominal sepsis treated non-operatively
Follow-up and outcomes
• +8 months: left lateral segmentectomy of the liver, resection of multiple liver nodules • +20 months: solitary liver lesion treated with percutaneous RFA • +35 months: multiple liver lesions, open resection with intraoperative RFA • +52 months: Percutaneous RFA of central liver lesion • +77 months: Negative octreotide scan, percutaneous RFA of central liver lesion • +84 months: No evidence of disease, monthly administration of long acting release somatostatin (20 mg) and diabetes mellitus (treated with insulin)

*CT* Computed tomography, *RFA* radiofrequency ablation

Metastases to the spleen have been reported from a variety of primary tumors. They remain extremely rare, no matter what the primary lesion is. In a meta-analysis of 713 patients with splenic metastases, Lam reported 23% from breast cancer primaries, 20% from lung, 9% from colon/rectum, 9% from ovary and the rest from the stomach, prostate, and other organs [6]. The reported incidence may be increasing due to improvements in imaging technology, but they are usually found in patients with multiple visceral metastatic sites in the terminal stage [7]. Solitary splenic metastases are very unusual. The pathogenesis of metastases to the spleen is usually considered to be of arterial origin, but may be from tumor thrombi in the splenic vein [8]. The reasons for the rarity of splenic metastases rare are not understood. Mechanical factors may limit implantation of cells (angle of the splenic artery and contraction of the organ). There are also micro-environmental factors (absence of afferent lymphatics and local anti-tumor activity) which inhibit the growth of metastatic cells [6, 7]*.*

We report the case of a patient who initially presented with a pNET with synchronous liver metastases and a single metastasis to the spleen. This is the first report of a metastasis to the spleen from a pNET.

## Case presentation

**Fig. 1:**
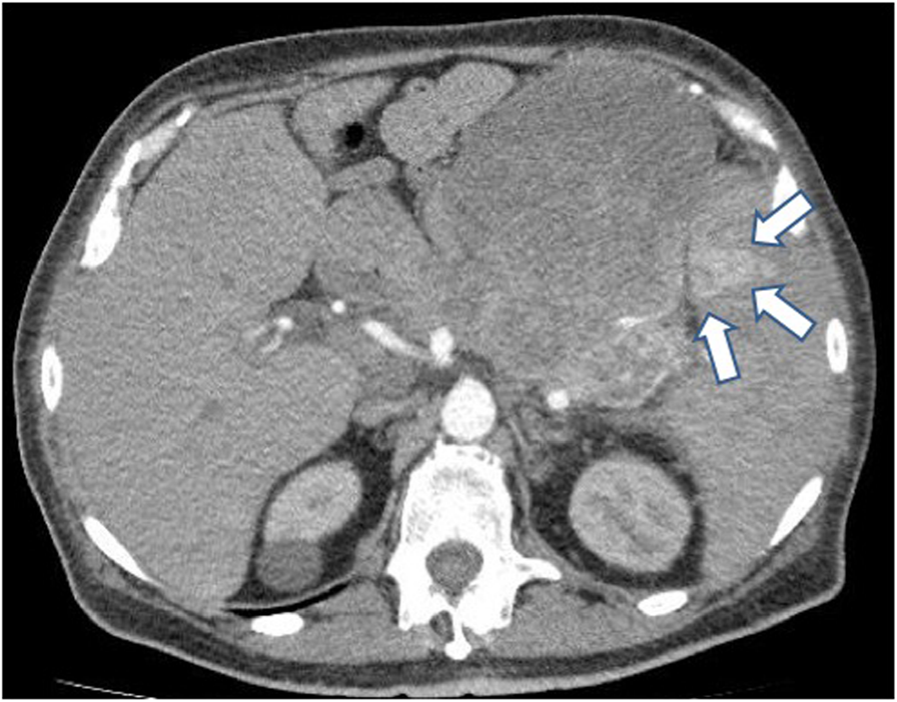
Preoperative computed tomography scan of the abdomen shows a 24 cm mass in the left upper quadrant between the spleen and liver, and a single metastatic lesion (1 cm) in the spleen (*arrows*)

The patient presented as a 53 years old white male with anemia and fatigue. Previous history included no medications, distal left lower extremity venous thrombophlebitis 2 months prior, status post laparoscopic gastric Roux-en-Y bypass (for obesity and diabetes mellitus which had been untreated and resolved postoperatively) three years prior to presentation and a left ulnar nerve decompression nine years prior. Physical examination revealed no palpable lymphadenopathy, a fullness in the left upper quadrant and fecal occult blood positive stool. Laboratory studies showed a serum hemoglobin of 6 mg/dl. Computed tomography (CT) scan of the abdomen showed a 24 cm left upper quadrant mass with multiple liver lesions, splenomegaly and a 1 cm mass in the spleen (Fig. 1). A laparoscopic liver biopsy was performed and histopathology was initially reported as hepatocellular carcinoma versus hepatoid carcinoma. One month later, serum hemoglobin was 4.4 mg/dl and gross melena was seen. Blood transfusions did not resolve the anemia. Serum albumin was decreased to 1.7 g/dl.

**Fig. 2:**
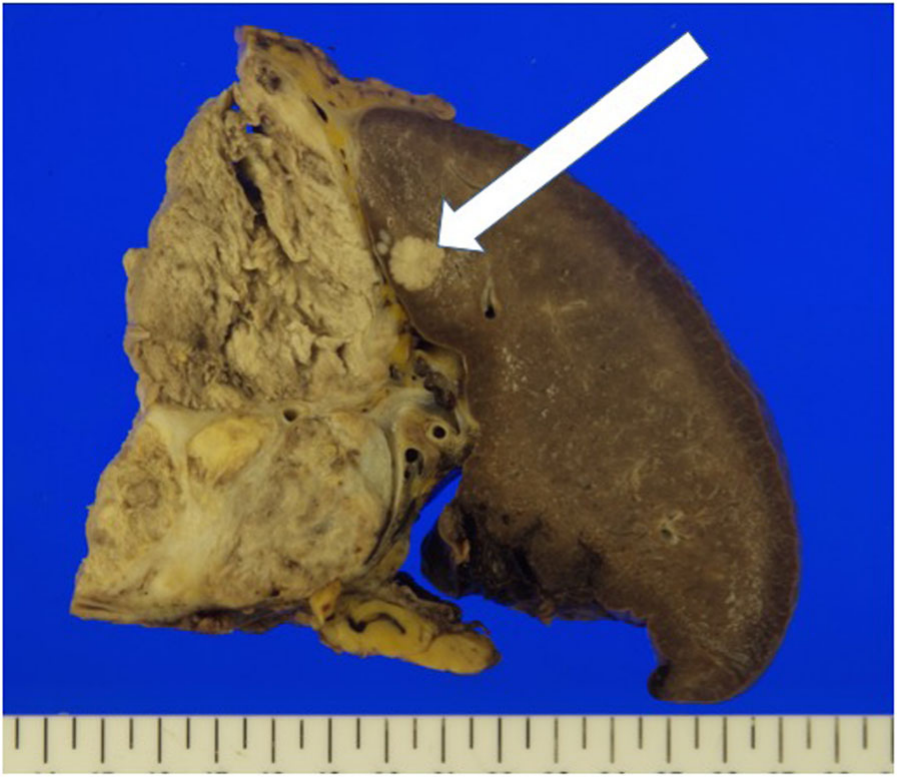
Gross pathology image of the cut surface of the spleen and tumor showing a 1.0 cm metastasis (arrow), corresponding to the lesion seen on the computed tomography scan

**Fig. 3:**
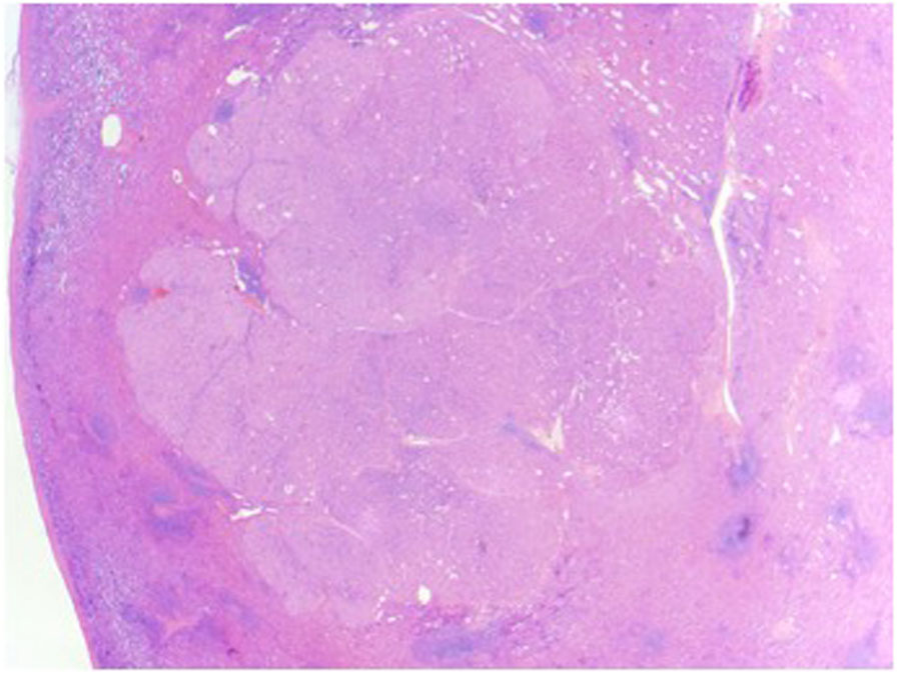
Histopathology of the spleen lesion, with an appearance similar to the histopathology of the primary lesion in the pancreas. The metastatic lesion extends close to, but not through the splenic capsule and is separate from the adjacent tumor. (Hematoxylin and eosin stain, 15X)

The patient underwent gastrectomy, splenectomy, distal pancreatectomy and resection of small bowel and a portion of colon that were adherent to the tumor mass. At the end of surgery, there was no gross tumor remaining except for the previously identified liver metastases. Pathology showed a tumor that originated in the pancreas with direct extension to the small bowel, colon, and stomach. The resected specimen weighed 3500 g and the spleen measured 14x7x10cm (Fig. 2). On cut section, the spleen contained a 1.0 cm lesion consistent with a metastasis, which was then confirmed to be a metastatic lesion on histopathologic evaluation (Fig. 3).

**Fig. 4:**
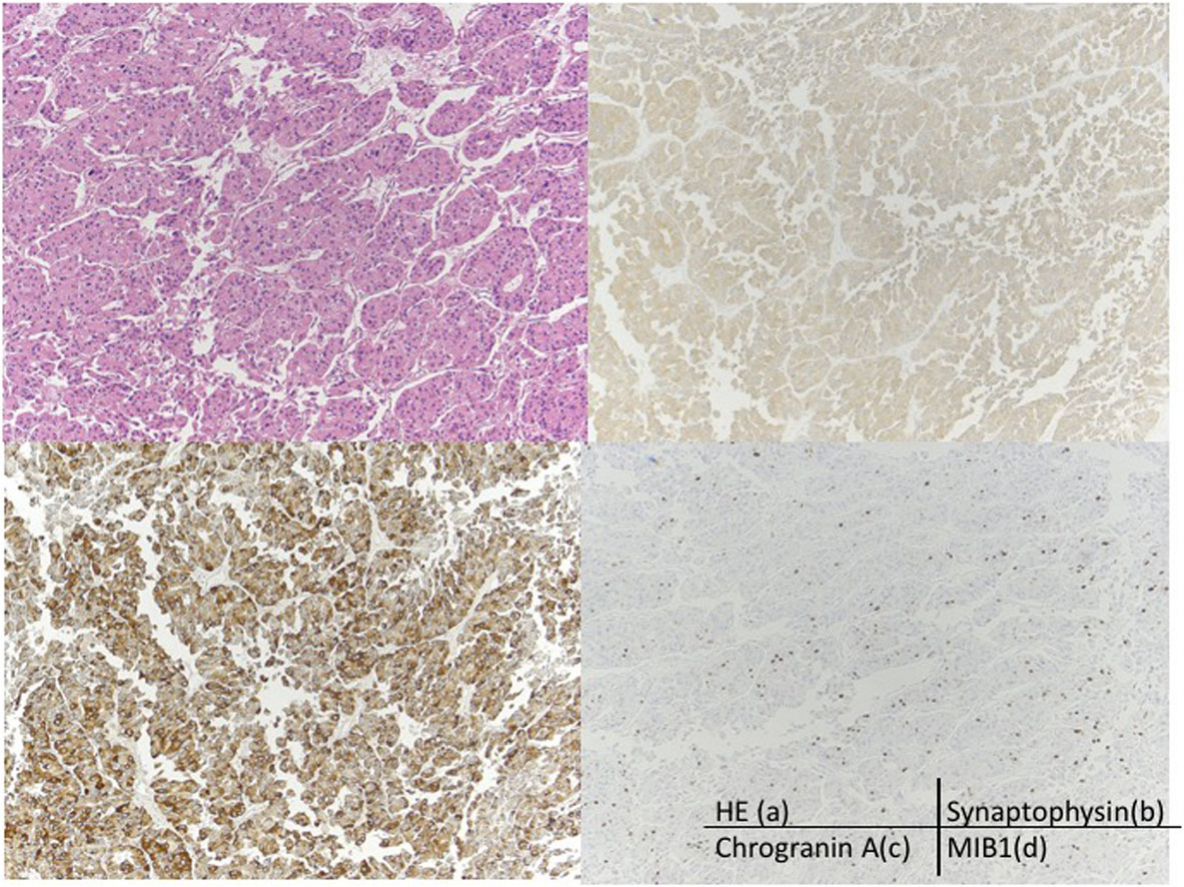
**a** Histopathological findings revealed that the tumor was composed of cells with round nuclei arranged in sheets or rosettes (Hematoxylin and eosin stain × 100). **b** The tumor cells were weakly positive for synaptophysin by immunohistochemical staining (×100), **c** The chromogranin A (×100), and MIB 1 index was about 10% (**d**) In this case, CD56 and other immunohistochemical stains were negative except for SSTR-2 (x100)

The final pathology report showed a pNET, well differentiated, stage T4N0M1 with 0/13 lymph nodes containing tumor. The lesion in the spleen was located near the edge of the spleen, but there was no direct invasion by the tumor mass (Figs. 2 and 3) grossly or histologically. Examination of the splenic vein showed no evidence of thrombosis with only minimal invasion by the tumor histologically. There were three mitoses per 50 high power fields, and Ki-67 < 1%. Perineural and lymphovascular invasion was seen. Immunostains for chromogranin A and synaptophysin are positive (Fig. 4). Immunostains for CD56, OCGH1E5, AFP, glucagon, insulin, somatostatin, pancreatic polypeptide, alpha 1-anti trypsin, alpha 1 anti-chymotrypsin and lipase are negative. Blood chemistry studies showed that this was a non-functional tumor.

The patient was discharged home ten days after surgery and returned two weeks later with intra-abdominal sepsis treated with drainage of fluid and antibiotics. Diabetes mellitus recurred in this patient after pancreatic resection. Somatostatin administration, long-acting release 20 mg/month was started. Approximately 8 months after the resection of the primary lesion, repeat imaging studies (CT scan, magnetic resonance imaging) and an octreotide nucleotide scan showed no lesions outside the liver. Left lateral segmentectomy was performed with simultaneous resection of multiple hepatic nodules throughout the liver. All lesions were consistent with metastases from the previously resected primary tumor on histopathologic examination.

The patient was discharged without complications, and continued monthly somatostatin therapy as well as periodic CT scans to evaluate recurrence. About one year after liver resection, a new lesion appeared in the liver which was treated with percutaneous radiofrequency ablation (RFA). The patient did well until 14 months later when multiple liver lesions appeared. A second liver resection was completed, including resection of nine nodules (<1 cm size) and RFA of two central liver lesions. It was felt that all lesions visible were treated at that time. Seventeen months later, another liver lesion appeared which was centrally located and treated with percutaneous RFA. An octreotide scan showed no evidence of disease. About 20 months later another central area in the liver was treated with percutaneous RFA.

The patient is now seven-years status post resection of the primary lesion, treated with monthly administration of somatostatin long acting release 20 mg IM, and followed with periodic CT scans of the liver. Diabetes mellitus persists and is treated with insulin. At seven years, there is no evidence of residual disease in the abdomen, or within the parenchyma of the liver. He remains asymptomatic and is doing well. The patient’s clinical history is summarized in the Table 1, prepared in accordance with CARE guidelines [9].

## Discussion

Neuroendocrine tumors are rare, but their reported incidence has increased over the last decades, which is attributed to improved diagnostic modalities and surveillance [4, 7]. In one study, the incidence from 1974 to 2004 more than doubled, from 1.4 new cases per million to 3.0 [10]. Gastrointestinal neuroendocrine tumors have a higher likelihood of presenting with metastases, which are present in about 50–65% of patients at the time of diagnosis. With the availability of new treatment modalities, long-term survival is reported, even in patients who present with metastatic disease. A recent review showed that the combination of long-acting somatostatin analogue therapy with aggressive cytoreductive surgery can result in long-term survival in some patients with metastatic NETs originating in the gastrointestinal tract [4].

In a series of 46 patients with pNETs, 48% presented with loco-regional disease and 52% presented with metastases [11]. All patients without metastases underwent resection, and the authors report a three-year survival of 86% with a median 42 months follow-up. Patients with metastatic disease were treated with a variety of modalities including resection, ablation, chemoembolization and others, and had a three-year survival of 70%. The authors credit their use of a multidisciplinary multimodal approach. An aggressive approach to these lesions was also reported by Touzios et al [1], who categorized the treatment of 60 patients with pNET into three groups: no treatment, resection, and transarterial chemoembolization. Five-year survival was 25% in the no treatment group, 72% for the resection group and 50% for the transarterial treated group. They also found poor outcomes in patients with >50% involvement of the liver.

The impact of lymph node involvement on survival in patients with pNET was studied by Krampitz and colleagues [12]. They found that disease-related survival decreased as a function of the number of lymph nodes involved. However, in patients with liver metastases and lymph node metastases, the major determinant of survival is the presence of liver metastases. The probability of survival at 10 years is 30% in this series. In a large series of patients with non-functional pNETs, tumor size and nodal status were not associated with survival, while tumor grade and systemic metastases were associated [10].

Somatostatin receptor scintigraphy is an important modality in the evaluation of these patients, and peptide receptor radionuclide therapy has shown promising results in the therapy of these lesions [13]. Transplantation has been used in select patients with results similar to transplantation performed for hepatocellular carcinoma [14]. These authors advise waiting for stabilization of liver disease prior to proceeding to transplantation. However, transplantation is generally not considered as a first-line therapy for metastatic neuroendocrine tumors [4].

The presence of splenic metastases is usually reported in the context of a pre-terminal event with multiple visceral metastases. In this patient, it is of interest that the single spleen metastasis was found at the initial presentation. The origin of the splenic metastasis is the subject of some speculation. In the spleen, it is believed that reticulo-endothelial create an unfavorable environment for the growth and survival of tumor cells [6, 7, 15]. Splenic metastases are believed to originate from the splenic artery, splenic vein, or lymphatics [8]. However, there are few lymphatic ducts to the spleen, and most metastases are considered of hematogenous origin [16]. Marymont et al demonstrated that splenic metastases are seen in the venous sinusoids and /or red pulp, supporting a hematogenous origin [17]. The splenic artery would appear to be the most common origin of splenic metastases although this suggests systemic circulation of tumor cells, and metastases in other organs may be expected, making solitary splenic metastases less likely. In patients with pNET, liver metastases from the portal vein are extremely common. In the present patient, although histologic evaluation of the portal vein showed no evidence of portal vein thrombus, the huge tumor mass and minimal invasion of the splenic vein seen on histologic evaluation, may have affected portal vein outflow, leading to congestion of the splenic vein and possibly resulting in development of a splenic metastasis [18].

In patients with late appearance of splenic lesions, treatment may be futile but splenectomy may benefit patients who experience significant pain due to splenomegaly. Isolated splenic metastases were extremely rare in a series of 92 splenic metastases, representing just 4% of the lesions [6]. In that series, 26% of the spleens weighed more than 200 g. It is estimated that only about 100 cases of isolated splenic metastases have been reported [19], from diverse primary tumors. Splenectomy for isolated metastases can result in long term survival [7]. It has been suggested that splenic metastases may result from the growth of early blood-borne tumor cells, after a latent period [7]. Laparoscopic splenectomy is a reasonable surgical option in a patient with an isolated splenic metastasis.

Despite an extensive review of the literature including multiple databases (keywords “spleen”, “metastases”, “neuroendocrine”), there are no reports of patients with metastases to the spleen from pNETs. In one review of unusual locations for metastases from neuroendocrine tumors, there were no lesions metastatic to the spleen [20]. Metastases to the spleen have been reported for bronchial carcinoid tumors [21, 22]. The spleen has been reported to be involved with neuroendocrine tumors of the pancreas, including a tumor thrombus in the splenic vein without a mass in the spleen [23] and as a splenic mass from direct extension and gastric varices [24]. The rarity of splenic metastases is not entirely surprising, since pNETs and splenic metastases are both rare entities.

## Conclusions

This is the first report of a splenic metastasis from a primary pancreatic neuroendocrine tumor. There are a number of interesting features in the present patient’s clinical history. The spleen metastasis in this patient was solitary and found at initial presentation, rather than as a pre-terminal event. This patient had no lymph node metastases at initial presentation which is unusual in the presence of liver metastases. This patient has undergone aggressive cytoreductive treatment and monthly administration of long-acting somatostatin analogue that have likely contributed to the continued survival of this patient.

## Abbreviations

CT: Computed tomography
NET: Neuroendocrine tumor
pNET: Pancreatic neuroendocrine tumor
RFA: Radiofrequency ablation
